# The Eight-Hour Day—Myth or Reality

**Published:** 1920-10-16

**Authors:** 


### The Eight-Hour Day—Myth or Reality.

We have received a communication from one of the leading training-schools in the Provinces craving to know to what extent the fifty-six hour week has got beyond the board-room. That it has met with favourable consideration in every quarter and been determined on by managers and governors with a remarkable consensus of opinion is known to us all. But does this mean that it is generally in actual working order?

The impediments to starting and maintaining a forty-eight hour week are want of money and want of probationers. Everyone recognises that probationers will continue to fight shy of nursing until the eight-hour day becomes a reality, but then until probationers begin to pour in, the nurses are compelled to be on duty a good deal longer than anybody desires in order to get the work done. How is any escape to be found from this vicious circle?

If we add to these problems the crushing burden of finding money for the extra ten or fifteen per cent. of the staff required to bring the forty-eight hour week into being, it is not surprising if it still holds off in spite of all the resolutions in its favour. We do know of certain institutions which have carried it into effect. Undoubtedly it is just and a main factor in attracting the right kind of probationer to the Service. But even when once started it is not easy to keep it up, for the slightest fluctuation in the numbers of the staff, occasioned by illness or other causes, may cause it to break down. We should be grateful to any matron who could recount six months' experience of its practical working.

As regards the fifty-six hour week for which the College of Nursing contends, we believe there are now few institutions in which this is not on the road to realisation, though there are difficulties even here in certain localities. We wish we could add that the scale of salaries suggested by the College is as universally accepted as the fifty-six hour week.

